# Serum levels of per- and polyfluoroalkylated substances and methylation of DNA from peripheral blood

**DOI:** 10.3389/fpubh.2025.1621495

**Published:** 2025-07-28

**Authors:** Hanane Omichessan, Dzevka Dragic, Vittorio Perduca, Thérèse Truong, Silvia Polidoro, Marina Kvaskoff, German Cano-Sancho, Jean-Philippe Antignac, Laura Baglietto, Francesca Romana Mancini, Gianluca Severi

**Affiliations:** ^1^Université Paris-Saclay, UVSQ, Inserm, Gustave Roussy, CESP, Villejuif, France; ^2^Département de Médecine Sociale et Préventive, Faculté de Médecine, Université Laval, Québec, QC, Canada; ^3^Centre de Recherche sur le Cancer, Centre de Recherche du CHU de Québec - Université Laval, Axe Oncologie, Québec, QC, Canada; ^4^Université Paris Cité, CNRS, MAP5, Paris, France; ^5^Department of Translational Medicine, University of Eastern Piedmont, Novara, Italy; ^6^Oniris, INRAE, LABERCA, Nantes, France; ^7^Department of Clinical and Experimental Medicine, University of Pisa, Pisa, Italy; ^8^Department of Statistics, Computer Science, Applications "G. Parenti" (DISIA), University of Florence, Florence, Italy

**Keywords:** endocrine disrupting chemicals, environmental exposure, DNA methylation, PFOA, PFOS

## Abstract

**Background:**

Perfluorooctanoic acid (PFOA) and Perfluorooctane sulfonate (PFOS) are among numerous chemicals in the Per- and polyfluoroalkylated substances (PFAS) group, which are commonly present in various consumer and industrial products. These chemicals are recognized for their persistency, the ability to accumulate in biological systems and their documented adverse effects on human health. Previous research, which has primarily centered on global methylation patterns, has suggested that some effects of PFAS on human health may be linked to modifications in DNA methylation (DNAm). The aim of our study was to assess the relationship between the serum levels of PFOS and PFOA and CpG site-specific methylation of DNA from peripheral blood.

**Methods:**

We used a case–control study on breast cancer nested within the E3N cohort, a prospective study of French women, in which we measured DNAm at more than 850,000 CpG sites with the Illumina Infinium MethylationEPIC BeadChip for 166 case–control pairs. Serum levels of PFOS and PFOA were measured by liquid chromatography coupled to tandem mass spectrometry.

**Results:**

We found 64 CpG sites with significant hypomethylation or hypermethylation associated with increased levels of PFOA or PFOS (*p*-value_Bonferroni_ < 0.05). The strongest association was found between PFOA serum levels and decreased DNAm at cg06874740 (*p*-value_Bonferroni_ = 2.2×10^−5^) and between PFOS serum levels and decreased DNAm at cg02793158 (*p*-value_Bonferroni_ = 9.3×10^−5^). Gene-set enrichment analyses using all CpG sites associated with PFOA or PFOS with an unadjusted *p*-value <0.01, identified 20 KEGG pathways for each of these compounds.

**Conclusion:**

PFAS exposure may be linked to substantial and widespread changes in the methylome that may be involved in the consequences on health of these pollutants. Our findings indicate that the biological and health effects of PFOA and PFOS may be more intricate and varied than previously thought, reinforcing the need for policies aimed at regulating this class of endocrine-disrupting chemicals.

## 1 Background

Per- and polyfluoroalkylated substances (PFAS) represent an extensive array of chemical compounds, encompassing over 4,000 different congeners (1). These compounds exhibit various physical and chemical properties such as impermeability to grease, water and oil and resistance to heat. Because of these distinct properties, PFAS find extensive applications in various products, including in stain- and water-repellent textiles, carpets, cleaning products, paints and fire-fighting foams. Also, limited use in cookware and food packaging and processing had been authorized (U. S Food and Drug Administration).

Among all PFAS, perfluorooctanoic acid (PFOA) and perfluorooctanesulfonate (PFOS) have historically been the most commonly employed compounds. However, they are now prohibited and are undergoing close scrutiny and research regarding their impacts on human health and the environment. Both chemicals are sources of particular concern due to their ability to be transported over long distances, their persistence in the environment, and their capacity to accumulate gradually in living organisms (51). For these characteristics they are classified as persistent organic pollutants (POPs).

Exposure to these contaminants can occur through multiple pathways, and their lingering presence in the environment has resulted in escalating levels of environmental contamination arising from past and ongoing usage.

The growing concerns surrounding PFOA and PFOS exposure and their impact on human health have been underscored in a review conducted by Fenton et al. (2). Numerous studies have brought to light the fact that PFOA and PFOS can function as endocrine disruptors affecting immune function (3–5) and thyroid function (6), as well as reproductive and developmental outcomes (7–9). Furthermore, earlier research has identified an association between exposure to PFOS and PFOA and various health conditions, including liver and kidney diseases, and different types of cancers (2, 10, 11). For breast cancer (BC) in particular, a case–control study conducted among the Inuit population in Greenland found that elevated serum levels of PFOA and PFOS were linked to an increased risk of the disease (50); similar results were also observed in a case–control study nested within the French E3N cohort (12).

The precise mechanisms by which these substances operate are not yet fully comprehended. Nonetheless, there is a hypothesis that PFOA and PFOS may induce changes in DNA methylation (DNAm), a biochemical process involving the addition of a methyl (-CH_3_) group to the fifth carbon position of a DNA base, typically a cytosine base within a CpG dinucleotide. DNAm serves as a crucial epigenetic mechanism for regulating gene expression, and any alterations in this process could contribute to the development of various health conditions and diseases (47).

Studies conducted in-vitro, animal experiments, and human studies have identified several categories of environmental chemicals that may modify epigenetic marks such as DNAm. Such modifications have therefore been proposed as molecular markers of exposure to chemical and environmental agents (13). However, there is very limited research on the associations between exposure to PFOA and PFOS and DNAm at the level of single CpG sites in adults. Previous studies have examined associations between other PFAS compounds and DNAm among newborns or children (14), or global methylation marks such as LINE-1 and Alu elements (49). An exploratory study in 98 patients investigated the associations between plasma PFOA or PFOS and leukocyte DNAm and the mediating effect of DNAm on the PFOA/PFOS-blood lipid association. They found leukocyte DNAm alterations for 63 and 87 CpG sites as well as for 8 and 11 differentially methylated regions (DMRs) in relation to PFOA and PFOS serum levels, respectively (15).

We employed an approach similar to our prior research on brominated flame retardants (16) to investigate the association between serum levels of PFOA and PFOS and DNAm at individual CpG sites. We used DNA obtained from peripheral blood samples of a breast cancer case–control study nested in the E3N cohort. For this purpose, we conducted two epigenome-wide association studies (EWAS) to evaluate the association with PFOA and PFOS. Our underlying hypothesis is that alterations in DNAm can be used as indicators of exposure to PFAS.

## 2 Materials and methods

The aim of our study was to assess the relationship between the serum levels of PFOS and PFOA and CpG site-specific methylation of DNA from peripheral blood.

### 2.1 Population of interest

The *Etude Epidémiologique auprès de femmes de l’Education Nationale* (E3N) is a prospective cohort study that involves 98,995 French women born between 1925 and 1950, who at inclusion in 1990 were employed in the national education system and affiliated to the national health insurance MGEN (Mutuelle Générale de l’Education Nationale). Participation in this cohort study required women to return an initial self-administered questionnaire and provide explicit, informed and written consent. Subsequently, follow-up questionnaires were sent to participants every 2–3 years. These follow-up questionnaires gathered information about various aspects including lifestyle, dietary habits, medical history, and the use of medications and other treatments. A more comprehensive and detailed description of the E3N cohort can be found in two earlier publications (17, 18). This study obtained ethical approval from the French CNIL (Commission Nationale Informatique et Libertés).

We conducted a case–control study that comprised 166 BC cases and 166 controls nested within the E3N cohort. The female participants included in the case–control study were in the age-range of 47 to 72 years when their blood samples were collected, which took place from 1995 to 1998. The blood samples were divided into separate aliquots of buffy coat, plasma, serum and erythrocytes. For this study, cases and controls were individually matched based on factors such as age (within a 3-year range), body mass index (BMI), menopausal status, and the region of residence (specifically, the French departments) at the time of blood collection.

### 2.2 Measurement of PFAS circulating levels

Circulating levels of PFOA and PFOS were assessed by measuring them in serum samples through liquid chromatography coupled to tandem mass spectrometry (LC–MS/MS). To briefly describe the method that was previously detailed elsewhere (12), quantification was achieved according to the isotopic dilution method employing 13C labeled analogous as internal standards. The lipid content was determined with enzymatic kits provided by Biolabo (Maizy, France) independently for phospholipids (PL), triglycerides (TG), total cholesterol (TC) and free cholesterol (FC). The total serum lipids (TSL) were estimated using the formula proposed by Akins and colleagues: TSL = 1.677*(TC − FC) + FC + TG + PL (19).

The entire protocol adhered to well-established and accredited procedures, as outlined in the 2002/657/CE decision and compliant with the ISO 17025 standard. The quantification of PFOA and PFOS levels was conducted in nanograms per milliliter (ng/mL).

### 2.3 DNA methylation measurement and data pre-processing

The Infinium MethylationEPIC array, capable of quantifying DNAm in over 850,000 CpG sites, was employed to analyze DNA extracted from the archived buffy coats in a group of 197 BC case–control pairs.

The whole process, including DNA extraction, bisulfite conversion, quality control assessments, methylation assays, and data preprocessing was carried out at the Italian Institute of Genomic Medicine (IIGM) in Turin, Italy. They followed established procedures and methods previously developed by IIGM for prior studies on DNA methylation (20, 21). In addition, patients with low bisulfite conversion intensity, those with more than 5% missing values and the remaining unmatching pairs were removed.

After pre-processing, the final dataset consisted of 166 case–control pairs, and it included methylation data on 805,837 CpG sites. To conduct the association studies, M-values were utilized. For each CpG site, the M-value was determined as the log2 ratio of the intensities of the methylated probe to the unmethylated probe.

### 2.4 Cellular heterogeneity

Cellular composition is known to differ among individuals and since methylation levels at specific CpG sites vary with the type of cell, it is necessary to adjust for the proportion of cell types. Following the approach introduced by Houseman et al. (22), we utilized the methylation data to estimate the proportions of various cell types in each blood sample. These cell types included B cells, CD4 + T cells, CD8 + T cells, granulocytes, monocytes and natural killer cells.

### 2.5 Main statistical analyses

We examined whether DNAm were associated with the serum levels of PFOA and PFOS using linear mixed-effects models with the DNAm levels of individual CpG sites serving as the response variable. The circulating levels of PFOA (PFOS, respectively) were categorized according to the quartiles in the control group and the standardized median level within quartiles was considered as a pseudocontinuous fixed-effect. Additionally, we included array-related factors (plate and chips) as random effects to account for the variability in methylation due to technical sources. Furthermore, we adjusted our models for several covariates, which encompassed BC case–control status, age at blood collection (categorized as below or equal to the median value of 56.1 years or above the median value), parity and total breastfeeding duration (categorized as no children and no breastfeeding, at least 1 child and ≤6 months breastfeeding, at least 1 child and >6 months breastfeeding), BMI (categorized as below or equal 25 kg/ $m^{2}$ or above 25 kg/ $m^{2}$ ), proportions of different cell types, and lipids levels, defined in ng/mL and categorized based on the median value (≤6.46 and >6.46).

To account for the original case–control study design from which the data have been generated, we introduced weighting to the observations. This weighting was based on the prevalence of breast cancer in France, ensuring that the weighted data reflects the proportion of cases in the general population in line with an approach outlined by van der Laan (23). We performed our modeling using the *nlme* package in R. To address the issue of multiple testing we employed two methods. Firstly, we controlled the Family Wise Error Rate (FWER) by using Bonferroni adjusted *p*-values. Secondly, we controlled the False Discovery Rate (FDR) by calculating q-values with the *qvalue* R package (24). We considered all tests with FDR qvalues less than 0.05 to be statistically significant.

### 2.6 Sensitivity analysis

As sensitivity analyses we ran our models exclusively on the control group focusing only on women that have not been diagnosed with BC at the date of diagnosis of the matched case rather than using both cases and controls as described above.

### 2.7 Pathway analysis

After examining the relationship between PFOA and PFOS serum levels and DNAm, we carried out gene set analyses on the differentially methylated CpG sites. This analysis was performed using the *GOmeth* function from the *missMethyl* package (25). We focused on CpG sites that met the threshold for FDR-adjusted *p*-value of less than 0.05 in the association study. Each CpG site was mapped to genes using the IlluminaHumanMethylationEPICanno.ilm10b4.hg19 annotation package. A gene was considered differentially methylated if at least one CpG site within that gene exhibited significant differential methylation. The *GOmeth* method performs enrichment analysis of gene sets while correcting for two biases in methylation array data: “probe-number” (the number of CpG sites per gene present on the array) and “multi-gene” (CpGs associated with multiple genes) (25). For our analyses, we utilized the Kyoto Encyclopedia of Genes and Genomes (KEGG) collection pathways. A pathway was considered significantly enriched when genes within it were differentially methylated. As sensitivity analyses, we took the wider set of CpG sites associated with exposure to PFAS with unadjusted *p*-value <0.01. Pathway analyses were performed using R software version 4.1.2.

## 3 Results

The characteristics of the 332 women included in the study are presented in Table 1. The median age of the study participants at inclusion was 56.1 years, with one in four falling into the overweight or obese category. Approximately 39% of the women had never given birth or breastfed, while 40% had at least one child and breastfed for less than 6 months. The remaining 20% had a history of breastfeeding for more than 6 months.

**Table 1:** Baseline characteristics of the studied population and distribution of PFOS and PFOA concentrations in serum (*N* = 332).

		*N* = 332
**Patients baseline characteristics**		
Age (years), median (IQR)		56.10 (9.42)
BMI (kg/ $m^{2}$ ), mean (SD)		23.7 (3.39)
Parity, N (%)		
Nulliparous and no breastfeeding		131 (39.5)
Parous with less than 6 months of lactation		134 (40.3)
Parous with more than 6 months of lactation		67 (20.2)
Total lipids, median (IQR)		6.46. (1.23)
**Distribution of PFOS and PFOA concentrations in serum**		
PFOA (ng/g of mL)	median (IQR)	6.6 (3.73)
	mean (min, max)	7.3 (1.29, 21.39)
PFOS (ng/g of mL)	median (IQR)	17.39 (8.48)
	mean (min, max)	19.04 (5.84, 85.26)

Among the studied congeners, PFOS was the primary one, with a median serum concentration of 17.4 ng/mL. However, there was a substantial range in PFOS levels among women spanning from 5.84 ng/mL to 85.26 ng/mL, as detailed in Table 1. In the case of PFOA, the median concentration was 6.6 ng/mL, with a range of 1.29 ng/mL to 21.39 ng/mL. It is worth noting that there was a moderate correlation between the concentration levels of PFOS and PFOA (47%).

**Figure 1:**
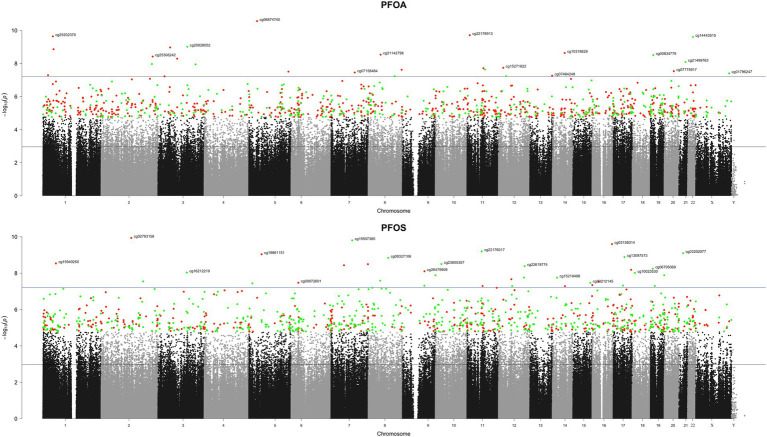
Manhattan plot of the 805,837 CpG sites in our EWAS analysis of PFAS congeners and DNA methylation. The y-axis is the −log10 *p*-value of the tests of associations between DNA methylation and circulating levels of PFAS. The black (resp. blue) line corresponds to a FDR (resp. FWER) threshold of 5%. Green and red colors represent the top 1,000, respectively, hyper- and hypo-methylated CpGs.

We identified 28 and 36 CpG sites, respectively, associated with serum levels of PFOA and PFOS at a genome-wide level of significance (Bonferroni adjusted *p*-value <5%), see Figure 1.

For each congener, the top CpG sites and their corresponding genes ranked according to their *p*-values are shown in Supplementary Tables S1, S2. In these tables, the estimates indicate the changes in DNAm associated with an increase of one unit of the pseudocontinuous PFOA (and PFOS, respectively) exposure variable. The top CpG position is cg06874740 located in *RAI14* (*p*-value = 2.20 × 10^−5^) for PFOA-related analysis and cg02793158 located in *LIMS2* (*p*-value = 9.31 × 10^−5^) for PFOS-related analysis.

**Figure 2:**
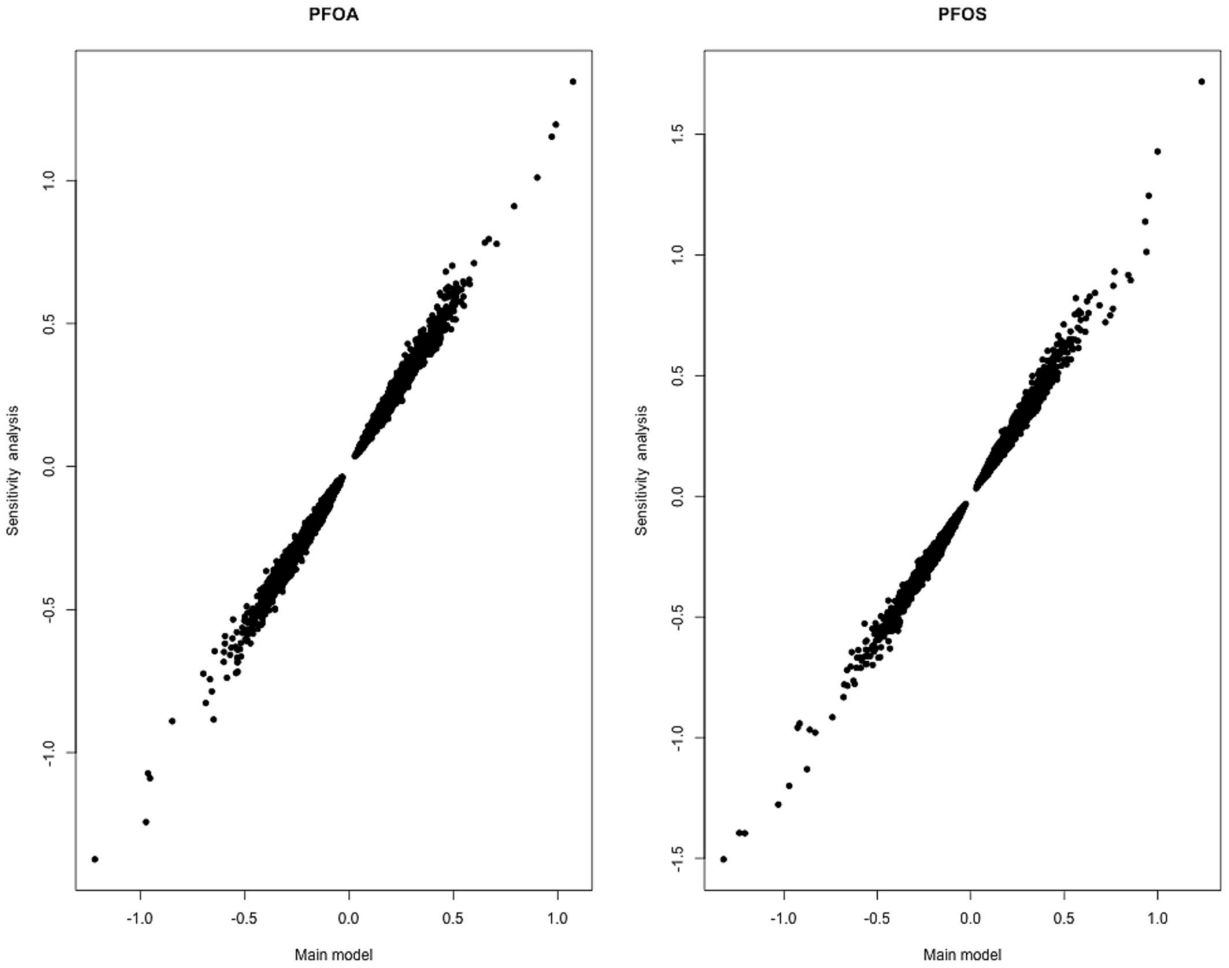
Comparison between the estimates of the PFOA coefficients (left) and the PFOS coefficients (right) in the linear mixed-effects models explaining DNAm levels of individual CpGs in the main analysis (x-axis) and the sensitivity analysis (y-axis). The main analysis includes both cases and controls, and the sensitivity analysis includes controls only. Each dot corresponds to a CpG site.

As shown in Figure 2, the estimates of the PFOA and PFOS coefficients in our main models fitted on BC cases and controls were consistent with the estimates in the models fitted on controls only.

**Figure 3:**
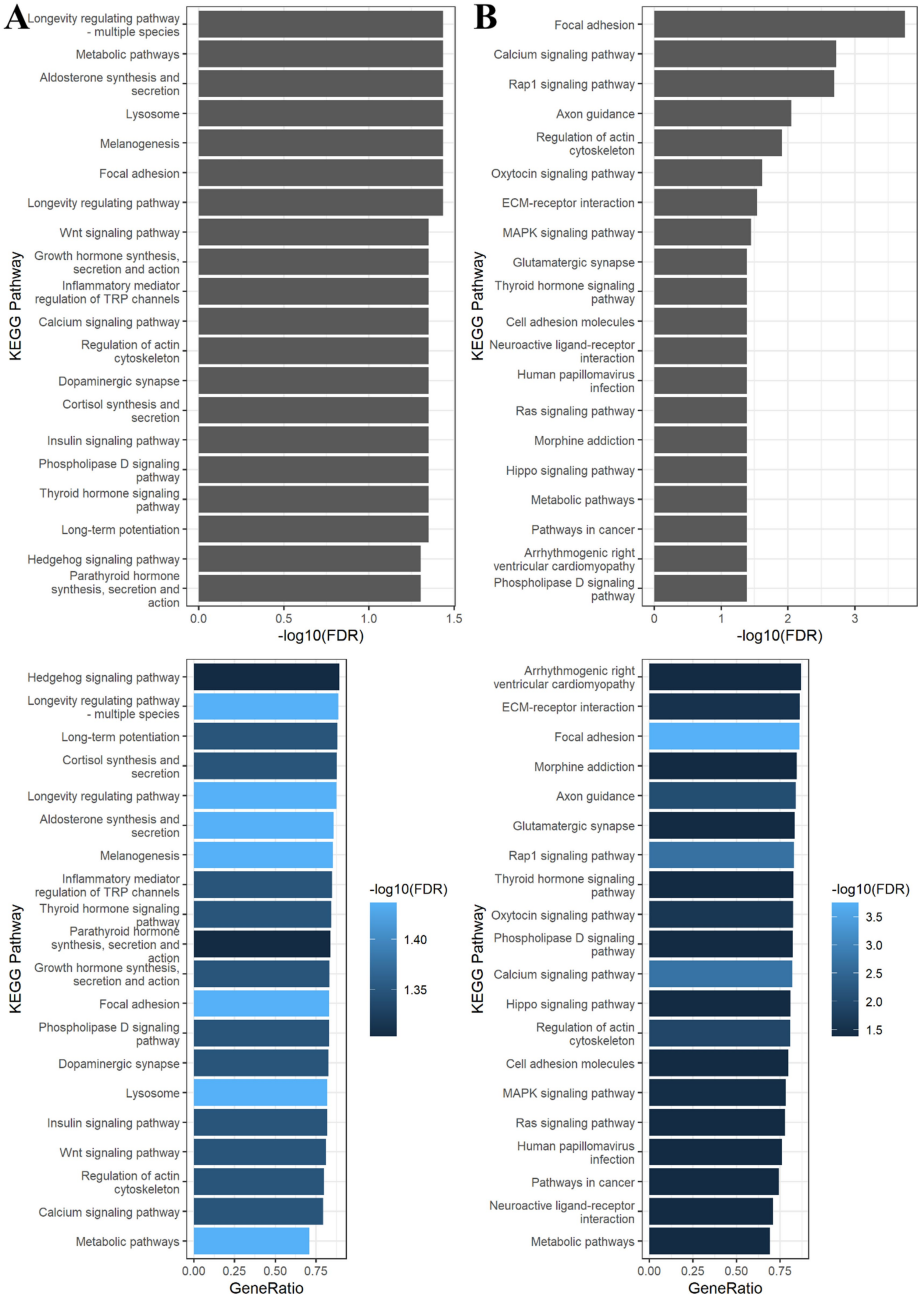
Bar charts for KEGG enrichment analysis of differentially methylated CpG sites associated with **(A)** PFOA and **(B)** PFOS at threshold unadjusted *p*-value <0.01 in the epigenome-wide association analyses. Pathways were considered significant when the FDR < 0.05. The terms of the KEGG pathways are depicted on the y-axis. On the top figure, the x-axis is the -log10 FDR of the tests of the gene set enrichment in each pathway. On the bottom figure, the x-axis is the ratio between the number of differentially methylated genes and the number of genes in the KEGG term. The different colors represent the −log10 FDR.

Pathway enrichment analyses using PFOA or PFOS-associated CpG sites from the EWAS analysis (FDR < 0.05; PFOA *n* = 12,414 located in 6,671 genes; PFOS *n* = 11,878 located in 6,298 genes) did not highlight any pathways in the KEGG collection. The sensitivity analyses using PFOA or PFOS-associated CpG sites with an unadjusted *p*-value <0.01 (PFOA *n* = 47,287 located in 14,928 genes; PFOS *n* = 46,116 located in 14,558 genes) identified 20 KEGG pathways for each compound (Figures 3A,B).

As shown in Table 2A, the results suggest that serum concentrations of PFOA may be associated with methylation changes in genes over-represented in pathways relative to the endocrine system (Thyroid hormone signaling pathway; Parathyroid hormone synthesis, secretion and action; Growth hormone synthesis, secretion and action; Melanogenesis; Aldosterone synthesis and secretion; Cortisol synthesis and secretion; Insulin signaling pathway), in signal transduction (Wnt signaling pathway; Calcium signaling pathway; Phospholipase D signaling pathway; Hedgehog signaling pathway), and in metabolic pathways.

**Table 2:** KEGG enrichment analysis of differentially methylated CpG sites associated with (A) PFOA and (B) PFOS at threshold unadjusted *p*-value <0.01 in the epigenome-wide association analyses.

(A) PFOA					
Classification of the KEGG pathway maps	Description	Number of genes in the KEGG term	Number of genes that are differentially methylated	*p*-value	FDR
Organismal Systems > Aging	Longevity regulating pathway	89	78	1.85 × 10^-04^	3.66 × 10^-02^
Cellular Processes > Cellular community - eukaryotes	Focal adhesion	200	166	3.29 × 10^-04^	3.66 × 10^-02^
Organismal Systems > Endocrine system	Melanogenesis	101	86	4.27 × 10^-04^	3.66 × 10^-02^
Cellular Processes > Transport and catabolism	Lysosome	132	108	5.90 × 10^-04^	3.66 × 10^-02^
Organismal Systems > Endocrine system	Aldosterone synthesis and secretion	98	84	6.94 × 10^-04^	3.66 × 10^-02^
Metabolism > Global and overview maps	Metabolic pathways	1,518	1,076	7.07 × 10^-04^	3.66 × 10^-02^
Organismal Systems > Aging	Longevity regulating pathway - multiple species	62	55	7.39 × 10^-04^	3.66 × 10^-02^
Organismal Systems > Nervous system	Long-term potentiation	67	59	1.04 × 10^-03^	4.48 × 10^-02^
Organismal Systems > Endocrine system	Thyroid hormone signaling pathway	121	102	1.20 × 10^-03^	4.48 × 10^-02^
Environmental Information Processing > Signal transduction	Phospholipase D signaling pathway	147	122	1.55 × 10^-03^	4.48 × 10^-02^
Organismal Systems > Endocrine system	Insulin signaling pathway	137	112	1.60 × 10^-03^	4.48 × 10^-02^
Organismal Systems > Endocrine system	Cortisol synthesis and secretion	65	57	1.67 × 10^-03^	4.48 × 10^-02^
Organismal Systems > Nervous system	Dopaminergic synapse	131	108	1.87 × 10^-03^	4.48 × 10^-02^
Cellular Processes > Cell motility	Regulation of actin cytoskeleton	217	173	1.97 × 10^-03^	4.48 × 10^-02^
Environmental Information Processing > Signal transduction	Calcium signaling pathway	238	189	2.00 × 10^-03^	4.48 × 10^-02^
Organismal Systems > Sensory system	Inflammatory mediator regulation of TRP channels	98	83	2.19 × 10^-03^	4.48 × 10^-02^
Organismal Systems > Endocrine system	Growth hormone synthesis, secretion and action	119	99	2.30 × 10^-03^	4.48 × 10^-02^
Environmental Information Processing > Signal transduction	Wnt signaling pathway	169	137	2.32 × 10^-03^	4.48 × 10^-02^
Organismal Systems > Endocrine system	Parathyroid hormone synthesis, secretion and action	106	89	2.73 × 10^-03^	4.97 × 10^-02^
Environmental Information Processing > Signal transduction	Hedgehog signaling pathway	56	50	2.86 × 10^-03^	4.97 × 10^-02^

(B) PFOS					
Classification of the KEGG pathway maps	KEGG pathway	Number of genes in the KEGG term	Number of genes that are differentially methylated	*p*-value	FDR
Cellular Processes > Cellular community - eukaryotes	Focal adhesion	200	172	5.12 × 10^-07^	1.78 × 10^-04^
Environmental Information Processing > Signal transduction	Calcium signaling pathway	238	195	1.09 × 10^-05^	1.89 × 10^-03^
Environmental Information Processing > Signal transduction	Rap1 signaling pathway	210	174	1.76 × 10^-05^	2.03 × 10^-03^
Organismal Systems > Development and regeneration	Axon guidance	181	152	1.02 × 10^-04^	8.88 × 10^-03^
Cellular Processes > Cell motility	Regulation of actin cytoskeleton	217	175	1.77 × 10^-04^	1.23 × 10^-02^
Organismal Systems > Endocrine system	Oxytocin signaling pathway	154	127	4.21 × 10^-04^	2.44 × 10^-02^
Environmental Information Processing > Signaling molecules and interaction	ECM-receptor interaction	88	76	5.89 × 10^-04^	2.92 × 10^-02^
Environmental Information Processing > Signal transduction	MAPK signaling pathway	294	230	8.28 × 10^-04^	3.59 × 10^-02^
Environmental Information Processing > Signal transduction	Phospholipase D signaling pathway	147	121	1.24 × 10^-03^	4.11 × 10^-02^
Human Diseases > Cardiovascular disease	Arrhythmogenic right ventricular cardiomyopathy	77	67	1.31 × 10^-03^	4.11 × 10^-02^
Human Diseases > Cancer: overview	Pathways in cancer	528	393	1.33 × 10^-03^	4.11 × 10^-02^
Metabolism > Global and overview maps	Metabolic pathways	1,518	1,050	1.82 × 10-^-03^	4.11 × 10^-02^
Environmental Information Processing > Signal transduction	Hippo signaling pathway	157	127	1.92 × 10^-03^	4.11 × 10^-02^
Human Diseases > Substance dependence	Morphine addiction	91	77	1.96 × 10^-03^	4.11 × 10^-02^
Environmental Information Processing > Signal transduction	Ras signaling pathway	234	182	2.00 × 10^-03^	4.11 × 10^-02^
Human Diseases > Infectious disease: viral	Human papillomavirus infection	329	250	2.04 × 10^-03^	4.11 × 10^-02^
Environmental Information Processing > Signaling molecules and interaction	Neuroactive ligand-receptor interaction	358	254	2.04 × 10^-03^	4.11 × 10^-02^
Environmental Information Processing > Signaling molecules and interaction	Cell adhesion molecules	152	121	2.13 × 10^-03^	4.11 × 10^-02^
Organismal Systems > Endocrine system	Thyroid hormone signaling pathway	121	100	2.27 × 10^-03^	4.11 × 10^-02^
Organismal Systems > Nervous system	Glutamatergic synapse	114	95	2.37 × 10^-03^	4.11 × 10^-02^

Pathways were considered significant when the FDR < 0.05.

As shown in Table 2B, the results suggest that serum concentrations of PFOS may be associated with methylation changes in genes mainly involved in signal transduction (Calcium signaling pathway; Phospholipase D signaling pathway; MAPK signaling pathway; Rap1 signaling pathway; Ras signaling pathway; Hippo signaling pathway), in endocrine system (Thyroid hormone signaling pathway; Oxytocin signaling pathway), in cancer pathways, and in metabolic pathways.

## 4 Discussion

As for other POPs, PFOS and PFOA exposure is a global concern. In this study, we aimed to investigate whether changes in blood DNA methylation levels could serve as indicators of exposure to PFOA and PFOS. We conducted our research on a sample of 332 French women participating in the prospective E3N cohort.

Consistent with findings from previous studies, we observed a significant moderate correlation between circulating levels of PFOA and PFOS (15, 26). Our analyses yielded evidence supporting an association between serum levels of PFOA and PFOS and DNAm. We identified 64 CpG sites that displayed statistically significant associations between DNAm levels and the circulating levels of PFOA (28 CpGs sites including 10 positive and 18 negative associations) and PFOS (36 CpGs sites including 22 positive and 14 negative associations) after Bonferroni correction.

To further explore the biological relevance of these associations, we highlight below some plausible mechanisms by which PFAS exposure may influence BC risk through DNA methylation changes. The CpG site cg06874740, located within the RAI14 gene, showed a negative association with PFOA exposure, suggesting hypomethylation with increasing exposure levels. Given than high expression of RAI14 is positively correlated with the malignant progression of breast cancer and suggests a worse prognosis (27), this finding supports a potential epigenetic mechanism through which PFOA exposure could influence BC progression.

The CpG cg25202370, located within the HIVEP3 gene, was also significantly hypomethylated with increasing PFOA levels. Hypomethylation at this site may lead to increased HIVEP3 expression, potentially enhancing NF-κB pathway activity, which is frequently dysregulated in cancer (28). Regarding PFOS, the site cg02793158, located within LIMS2, was significantly hypomethylated. Since LIMS2 encodes a protein involved in cell adhesion and cytoskeletal pathways, this hypomethylation may potentially increase cell motility and invasion - key hallmarks of cancer metastasis. Although its role in BC is not well characterized, the function of LIMS2 suggests its dysregulation could contribute to tumor progression. The CpG site cg15507385, located within the CDK14 gene, showed significant hypermethylation with higher PFOS exposure. Given CDK14 is expressed in the mammary basal layer and is elevated in triple-negative breast cancer (29), it may contribute to tumor aggressiveness.

PFAS are known to act as endocrine disrupting chemicals, thus it is consistent that DNAm alterations associated with circulating levels of PFOA and PFOS are involved in the endocrine system. It has been shown that PFOS has the ability to act as an endocrine disruptor both *in vitro* and *in vivo* by disrupting the function of nuclear hormone receptors and altering the expression of endocrine-related genes in animal models (30). However, there are potentially many unknown mechanisms linking PFAS to health, and the high production volume of many unregulated PFAS highlights the need for new policies.

Some studies also investigated the genome-wide changes in DNAm caused by PFAS exposure in adults. Only four CpG positions reported in a recent study conducted among women achieved an association in our study (adjusted FDR < 0.05). Xu et al. (31) reported that cg23351738, cg27021181, cg07826657 and cg26071661, respectively annotated to *SNORA38*, *NET1*, *MAPKAP1* and *CARF* genes, were found differentially methylated. In our study, the first three were associated with PFOA exposure while the latter was associated with PFOS at FDR significance level. Only findings regarding cg23351738 and cg07826657 were consistent in terms of the direction of association. Most of these genes are protein coding genes and *NET1* has been associated with BC (genescards).

Associations reported in our study may not be replicated in previous studies, which may be related to the different designs and population. The specific present study design and populations prevent direct comparisons with previous studies that may explain little consistency. Regarding PFOA and PFOS concentrations, higher levels were found in our population (median values of 6.6 and 17.4 ng/mL respectively) compared to the similar study from Xu and colleagues (0.85 and 2.29 ng/mL respectively) (15) or data related to French Esteban study (2014–2016), 2.12 and 4.23, respectively, (52)

DNA methylation is an important mechanism through which environmental factors can impact an individual’s risk of disease, given its role in regulating gene expression (15). In our current study, we evaluated the possible involvement of PFOA and PFOS in various pathways. We accomplished this by conducting gene enrichment analyses of CpG sites associated with exposure to these substances.

While no KEGG pathway reached statistical significance based on CpGs passing an FDR cutoff of 0.05, an exploratory enrichment analysis was carried out using a more permissive threshold (unadjusted *p*-value <0.01) to highlight candidate pathways for hypothesis generation. The findings suggest that the serum concentrations of PFOA and PFOS may be linked to alterations in DNA methylation in genes mainly associated with the endocrine system, with signaling molecules and interactions, and signal transduction pathways. Interestingly, Miura et al. (32) have identified some of the pathways highlighted in our analysis for PFOS. These pathways include focal adhesion, axon guidance, oxytocin signaling pathway, ECM-receptor interaction, cell adhesion molecules, and the MAPK signaling pathway. It is worth noting that the MAPK signaling pathway plays a key role in cell proliferation, differentiation and migration. Alterations in this signaling cascade have been associated with tumorigenesis (48). Furthermore, Goodrich et al. (33) have reported an enrichment among the top differentially methylated genes for PFOS in Hippo signaling pathway and morphine addiction. Regarding pathway analyses for PFOA, Mirua et al. (32) also identified enrichment in lysosome and Wnt signaling pathway; and Cheng et al. (15) highlighted enrichment in dopaminergic synapse and thyroid hormone signaling pathway.

In a previous paper, we studied the association between Polybrominated Diphenyl Ethers (PBDEs) and Polybrominated Biphenyls (PBBs), as well suspected to act as Endocrine disrupting chemicals and classified as POPs, and DNAm using data from the same case–control study nested in the E3N cohort. Similarly, to current findings, we identified multiple (positive and negative) associations between PBDEs and PBBs and DNAm as well as potential alterations in hypoxia, glycolysis and adipogenesis.

Our study has multiple strengths including the discovery of DNAm positions associated with PFOA and PFOS exposure, providing new evidence for the elucidation of PFAS-induced DNA methylation changes in humans. To our knowledge, this is one of the first EWAS of PFOS and PFOA involving DNAm from circulating blood in adult women. To date, previous studies mostly focused on (1) other endocrine disrupting chemicals such as phthalates (34) or bisphenols (35, 36), (2) repetitive genomic elements that were used as markers of global methylation (i.e., Alu and LINE-1) or used cell lines, animal models and (3) newborn (37) or children cohorts (14, 38). In addition, our study measured DNAm in a more detailed manner with the use of the most recent microarray, the Illumina MethylationEPIC BeadChip with coverage of almost 1 million CpGs. This represents a coverage that is 6 times greater than the coverage of studies that used Alu and LINE-1 elements.

Additionally, our cohort study possesses a unique profile, representing women born between 1925 and 1950. As a result, it reflects historical and cumulative exposure to POPs during a period when production and release of these substances were at their peak.

However, our study has several notable limitations. First, it is cross-sectional in nature, meaning that measures in blood (i.e., PFOA and PFOS serum concentrations and DNA methylation) were all made from the same blood samples. Second, the sample size was relatively limited making the statistical power adequate only for moderate to strong associations. Furthermore, we cannot rule out that PFAS might influence DNA methylation in other target organs that were not accessible for this study. Given that individuals are often exposed to multiple chemicals simultaneously, some of which may share common sources of exposure (e.g., specific foods), it is possible that various other POPs may contribute to the observed results. Moreover, our study includes only women, aged between 47 and 72; therefore, it is not possible to conclude that our results can be extended to men, other age groups or non-European populations. This is a major limitation, considering that age (39), gender (40) and population differences (41–43) in DNA methylation have been documented in previous research. Another important consideration is that DNA methylation is tissue-specific. In our study, methylation was measured in peripheral blood leukocytes, which are frequently used as surrogate tissues in epigenetic epidemiology. However, methylation signatures can differ across tissue types, raising uncertainty about how accurately blood-based methylation reflects epigenetic alterations in other organs more directly involved in PFAS toxicity. For instance, studies have documented some discordance in methylation profiles between blood and internal tissues (44, 45), suggesting that the observed associations in blood may not be fully representative of effects in target organs. Given the known tissue-specific toxicokinetics of PFAS, particularly their bioaccumulation and effects in endocrine organs; it is important to interpret our findings with caution when extrapolating to potential health outcomes mediated through these tissues. Finally, we acknowledge that our study is observational, and thus, future mechanistic studies, such as those based on *in vitro* exposure models, are warranted to further validate our findings.

## 5 Conclusion

Our study has provided some initial evidence of an association between PFAS exposure and moderate to strong alterations in individual CpG sites in DNA from peripheral blood. Research on the impact of exposure to PFAS on epigenetic mechanisms is still relatively limited in human populations. While there is substantial evidence regarding the toxicity and health effects of PFOA and PFOS, the evidence linking the exposure to these substances and DNA methylation has remained somewhat scarce. In perspective, it would be interesting to replicate our study with other populations to assess the generalizability of our findings. Additionally, an interesting perspective for future studies is the development and application of models for mixtures of substances, such as the BKMR (46), to model and evaluate the complex relationships between methylation levels and a variety of compounds, including PFAS substances and other persistent organic pollutants.

In conclusion, the results from our study suggest that the health effects of PFOA and PFOS might be more intricate and diverse than initially anticipated. These findings lend support to policies and regulations aimed at controlling this class of endocrine disrupting chemicals. Moreover, future studies incorporating tissue-specific methylation analyses or multi-tissue comparisons are necessary to fully understand the systemic and organ-specific impact of PFAS exposure.

## Data Availability

The datasets for this article are not publicly available due to concerns regarding participant/patient anonymity. Requests to access the datasets should be directed to the corresponding author.
