# Correction: Serum levels of per- and polyfluoroalkylated substances and methylation of DNA from peripheral blood

**DOI:** 10.3389/fpubh.2025.1679534

**Published:** 2025-09-02

**Authors:** Hanane Omichessan, Dzevka Dragic, Vittorio Perduca, Thérèse Truong, Silvia Polidoro, Marina Kvaskoff, German Cano-Sancho, Jean-Philippe Antignac, Laura Baglietto, Francesca Romana Mancini, Gianluca Severi

**Affiliations:** ^1^Université Paris-Saclay, UVSQ, Inserm, Gustave Roussy, CESP, Villejuif, France; ^2^Département de Médecine Sociale et Préventive, Faculté de Médecine, Université Laval, Québec, QC, Canada; ^3^Centre de Recherche sur le Cancer, Centre de Recherche du CHU de Québec - Université Laval, Axe Oncologie, Québec, QC, Canada; ^4^Université Paris Cité, CNRS, MAP5, Paris, France; ^5^Department of Translational Medicine, University of Eastern Piedmont, Novara, Italy; ^6^Oniris, INRAE, LABERCA, Nantes, France; ^7^Department of Clinical and Experimental Medicine, University of Pisa, Pisa, Italy; ^8^Department of Statistics, Computer Science, Applications “G. Parenti” (DISIA), University of Florence, Florence, Italy

**Keywords:** endocrine disrupting chemicals, environmental exposure, DNA methylation, PFOA, PFOS

In the published article, there was an error in [Figure 3] as published. [Only the two panels corresponding to PFOS (3B) were displayed, while those related to Figure PFOA (3A) were missing]. The corrected [Figure 3] and its caption [ORIGINAL Caption] appear below.

**Figure 3:**
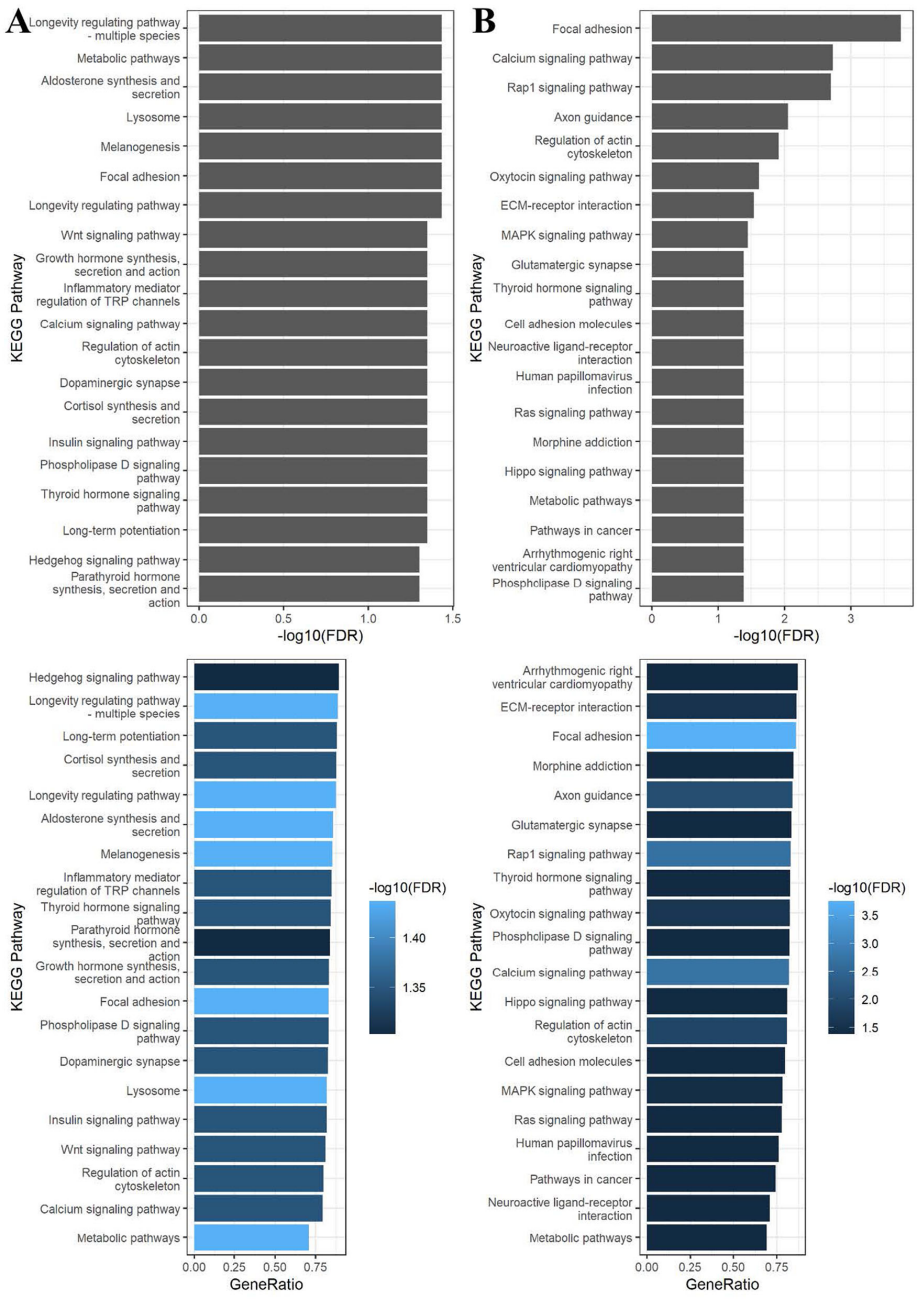
Bar charts for KEGG enrichment analysis of differentially methylated CpG sites associated with **(A)** PFOA and **(B)** PFOS at threshold unadjusted *p*-value < 0.01 in the epigenome-wide association analyses. Pathways were considered significant when the FDR < 0.05. The terms of the KEGG pathways are depicted on the *y*-axis. On the top figure, the *x*-axis is the –log10 FDR of the tests of the gene set enrichment in each pathway. On the bottom figure, the *x*-axis is the ratio between the number of differentially methylated genes and the number of genes in the KEGG term. The different colors represent the –log10 FDR.

The original article has been updated.

