# Supplementary material for: Serum levels of per- and polyfluoroalkylated substances and methylation of DNA from peripheral blood
Source: Front Public Health. 2025 Jul 28;13:1621495. doi: 10.3389/fpubh.2025.1621495 (PMC12336190; doi:10.3389/fpubh.2025.1621495)
Supplement: Supplementary file 1 [file Data_Sheet_1.pdf]

Supplementary table 1. Association between DNA methylation and PFOA

Probes	Estimates	SE	P	Bonferroni	UCSC gene
cg06874740	-3.3×10^{-1}	4.8×10^{-2}	2.7×10^{-11}	2.2×10^{-5}	RAI14
cg22176913	-4.5×10^{-1}	6.8×10^{-2}	1.9×10^{-10}	1.6×10^{-4}	
cg25202370	-1.3×10^{-1}	1.9×10^{-2}	2.3×10^{-10}	1.9×10^{-4}	HIVEP3
cg14443515	1.9×10^{-1}	2.9×10^{-2}	2.5×10^{-10}	2.0×10^{-4}	MAFF
cg20828052	2.9×10^{-1}	4.6×10^{-2}	9.8×10^{-10}	7.9×10^{-4}	OSBPL11
cg01066053	-1.3×10^{-1}	2.1×10^{-2}	1.1×10^{-9}	8.7×10^{-4}	IQCF3
cg05641624	-9.3×10^{-2}	1.5×10^{-2}	1.4×10^{-9}	1.1×10^{-3}	C1orf228
cg10319829	-4.0×10^{-1}	6.5×10^{-2}	2.3×10^{-9}	1.9×10^{-3}	
cg21142798	-1.6×10^{-1}	2.5×10^{-2}	2.9×10^{-9}	2.4×10^{-3}	PXDNL
cg00834779	2.9×10^{-1}	4.7×10^{-2}	3.1×10^{-9}	2.5×10^{-3}	ZNF700
cg25308242	-4.1×10^{-1}	6.7×10^{-2}	3.8×10^{-9}	3.1×10^{-3}	GLB1L
cg18993089	-2.1×10^{-1}	3.4×10^{-2}	5.1×10^{-9}	4.1×10^{-3}	
cg21499763	5.4×10^{-1}	9.1×10^{-2}	8.1×10^{-9}	6.5×10^{-3}	DSCR3
cg22860137	2.2×10^{-1}	3.8×10^{-2}	1.1×10^{-8}	8.7×10^{-3}	MREG
cg03765846	5.5×10^{-1}	9.3×10^{-2}	1.1×10^{-8}	9.2×10^{-3}	PPM1L
cg15271822	-3.1×10^{-1}	5.4×10^{-2}	1.8×10^{-8}	1.5×10^{-2}	AEBP2
cg00115821	-2.7×10^{-1}	4.7×10^{-2}	1.9×10^{-8}	1.6×10^{-2}	ORAOV1
cg02385173	2.1×10^{-1}	3.7×10^{-2}	2.3×10^{-8}	1.9×10^{-2}	ACER3
cg06319398	-2.7×10^{-1}	4.6×10^{-2}	2.4×10^{-8}	1.9×10^{-2}	
cg07775917	-1.6×10^{-1}	2.9×10^{-2}	2.9×10^{-8}	2.4×10^{-2}	PTPRT
cg26982433	-1.3×10^{-1}	2.2×10^{-2}	3.1×10^{-8}	2.5×10^{-2}	DOCK2
cg07156484	-8.7×10^{-2}	1.5×10^{-2}	3.5×10^{-8}	2.8×10^{-2}	PLOD3
cg01796247	1.9×10^{-1}	3.3×10^{-2}	4.0×10^{-8}	3.2×10^{-2}	SLITRK2
cg06705062	-2.7×10^{-1}	4.8×10^{-2}	5.1×10^{-8}	4.1×10^{-2}	ECE1
cg07464248	-2.3×10^{-1}	4.1×10^{-2}	5.5×10^{-8}	4.4×10^{-2}	TMCO3
cg12148263	3.4×10^{-1}	6.1×10^{-2}	5.8×10^{-8}	4.7×10^{-2}	METTL20
cg00828126	1.1×10^{-1}	1.9×10^{-2}	5.9×10^{-8}	4.7×10^{-2}	
cg14143723	-3.0×10^{-1}	5.4×10^{-2}	6.0×10^{-8}	4.9×10^{-2}	

Supplementary table 2. Association between DNA methylation and PFOS

Probes	Estimates	SE	P	Bonferroni	UCSC gene
cg02793158	-2.6×10^{-1}	3.8×10^{-2}	1.2×10^{-10}	9.3×10^{-5}	LIMS2
cg15507385	2.1×10^{-1}	3.1×10^{-2}	1.6×10^{-10}	1.3×10^{-4}	CDK14
cg03158314	-1.8×10^{-1}	2.8×10^{-2}	2.5×10^{-10}	2.0×10^{-4}	KIAA1609
cg22176017	1.5×10^{-1}	2.3×10^{-2}	6.3×10^{-10}	5.1×10^{-4}	POLR2G
cg03202077	1.8×10^{-1}	2.8×10^{-2}	8.0×10^{-10}	6.4×10^{-4}	ADAMTS5
cg19861151	-1.4×10^{-1}	2.1×10^{-2}	9.1×10^{-10}	7.4×10^{-4}	
cg13097573	2.1×10^{-1}	3.3×10^{-2}	1.3×10^{-9}	1.0×10^{-3}	
cg08327106	1.9×10^{-1}	3.0×10^{-2}	1.4×10^{-9}	1.2×10^{-3}	RALYL
cg15940250	-1.7×10^{-1}	2.8×10^{-2}	2.9×10^{-9}	2.3×10^{-3}	
cg23805357	1.7×10^{-1}	2.8×10^{-2}	3.2×10^{-9}	2.6×10^{-3}	LOC100128811, GPR158
cg25402818	-2.6×10^{-1}	4.2×10^{-2}	3.2×10^{-9}	2.6×10^{-3}	PTPRN2
cg15913831	-3.1×10^{-1}	5.1×10^{-2}	3.7×10^{-9}	3.0×10^{-3}	
cg22619774	6.9×10^{-1}	1.1×10^{-1}	4.1×10^{-9}	3.3×10^{-3}	PPP1CC
cg06795069	1.8×10^{-1}	3.0×10^{-2}	5.6×10^{-9}	4.5×10^{-3}	C19orf66
cg11554691	-2.0×10^{-1}	3.4×10^{-2}	6.5×10^{-9}	5.3×10^{-3}	TK1
cg26475909	-3.8×10^{-1}	6.4×10^{-2}	7.9×10^{-9}	6.4×10^{-3}	
cg16212219	5.5×10^{-1}	9.2×10^{-2}	9.3×10^{-9}	7.5×10^{-3}	MYLK
cg10023530	1.7×10^{-1}	2.8×10^{-2}	9.7×10^{-9}	7.8×10^{-3}	FAM38B
cg10296027	1.1×10^{-1}	1.8×10^{-2}	1.3×10^{-8}	1.0×10^{-2}	
cg11279918	2.6×10^{-1}	4.5×10^{-2}	1.3×10^{-8}	1.1×10^{-2}	ZNF544
cg24830367	1.4×10^{-1}	2.5×10^{-2}	1.7×10^{-8}	1.4×10^{-2}	PWP1
cg15218498	2.6×10^{-1}	4.4×10^{-2}	1.8×10^{-8}	1.4×10^{-2}	
cg02066277	-1.8×10^{-1}	3.2×10^{-2}	2.2×10^{-8}	1.7×10^{-2}	
cg00230631	2.4×10^{-1}	4.1×10^{-2}	2.6×10^{-8}	2.1×10^{-2}	SNTG1
cg25102782	1.5×10^{-1}	2.6×10^{-2}	2.8×10^{-8}	2.3×10^{-2}	PRKRA, DFNB59, MIR548N
cg07394965	-1.8×10^{-1}	3.1×10^{-2}	2.9×10^{-8}	2.4×10^{-2}	KIAA0556
cg09072601	-1.7×10^{-1}	3.0×10^{-2}	3.4×10^{-8}	2.7×10^{-2}	MDC1, TUBB
cg03212145	1.4×10^{-1}	2.5×10^{-2}	3.4×10^{-8}	2.8×10^{-2}	LOC101927153
cg17920099	7.6×10^{-1}	1.3×10^{-1}	3.7×10^{-8}	3.0×10^{-2}	FAM105B
cg04258138	-2.8×10^{-1}	5.0×10^{-2}	4.5×10^{-8}	3.6×10^{-2}	
cg03049782	1.9×10^{-1}	3.3×10^{-2}	4.8×10^{-8}	3.9×10^{-2}	TMEM106A
cg06132679	1.4×10^{-1}	2.4×10^{-2}	4.9×10^{-8}	4.0×10^{-2}	SYK
cg15073853	3.9×10^{-1}	6.9×10^{-2}	5.1×10^{-8}	4.1×10^{-2}	ISYNA1
cg12096894	-2.0×10^{-1}	3.6×10^{-2}	5.1×10^{-8}	4.1×10^{-2}	
cg15857731	4.0×10^{-1}	7.1×10^{-2}	5.1×10^{-8}	4.1×10^{-2}	CDK4
cg00715698	-2.5×10^{-1}	4.4×10^{-2}	5.2×10^{-8}	4.2×10^{-2}	RGS6
